# Supplementary material for: Cancer patient survival can be parametrized to improve trial precision and reveal time-dependent therapeutic effects
Source: Nat Commun. 2022 Feb 15;13:873. doi: 10.1038/s41467-022-28410-9 (PMC8847344; doi:10.1038/s41467-022-28410-9)
Supplement: Supplementary file 5 — Supplementary Dataset 2 [file 41467_2022_28410_MOESM5_ESM.zip › Supplementary Data File 2/Weibull all fits survival plots- Fig 1, 2, S1, S2/CA184043_2A_ipilimumab.pdf]

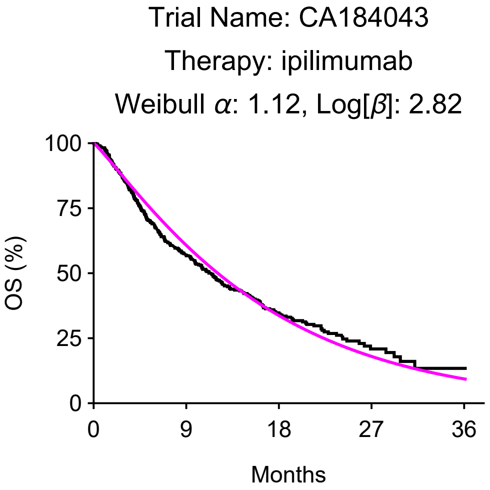
Trial Name: CA184043
Therapy: ipilimumab
Weibull α: 1.12, Log[β]: 2.82
100
75
50
25
0
OS (%)
0
9
18
27
36
Months
